# A network of bivalve chronologies from semi-enclosed seas

**DOI:** 10.1371/journal.pone.0220520

**Published:** 2019-07-30

**Authors:** Melita Peharda, Ivica Vilibić, Bryan Black, Hana Uvanović, Krešimir Markulin, Hrvoje Mihanović

**Affiliations:** 1 Institute of Oceanography and Fisheries, Split, Croatia; 2 Laboratory of Tree-Ring Research, The University of Arizona, Tucson, Arizona, United States of America; Union College, UNITED STATES

## Abstract

Four chronologies of the bivalve species *Glycymeris pilosa* have been constructed along a 300 km gradient of the eastern coastal Adriatic Sea, all of which span the common period of 1982–2015. The chronologies are compared to local and remote environmental drivers suspected to influence the biology of the system, including air and seawater temperature, precipitation and freshwater discharge. The Adriatic-Ionian Bimodal Oscillating System (BiOS), a key oceanographic feature quantified by satellite-derived absolute dynamic topography, is also compared to the chronologies. The chronologies at the two southern sites are more strongly influenced by local river discharge, while the two northern chronologies are more strongly influenced by BiOS. These results highlight the broadscale importance of BiOS to the Adriatic system as well as the heterogeneity of nearshore environmental and drivers of growth. These *G*. *pilosa* chronologies provide unique multidecadal, continuous, biological time series to better understand the ecology and fine-scale variability of the Adriatic with potential for other shallow, semi-enclosed seas.

## Introduction

Due to a lack of long-term instrumental records, proxies are necessary to establish historical ranges of environmental variability and provide context for assessing human influence on the climate system. Growth increment widths from biological archives provide one such proxy and have been used to develop absolutely dated, annually-resolved, and environmentally sensitive time series that extend from multiple decades to multiple millennia. Tree rings are the archetypal example, and the current network of datasets publicly available through the International Tree-Ring Databank now numbers more than 4,000. This network of chronologies has been used to assess long-term patterns of climate and disturbance in terrestrial settings at scales ranging from single stands to hemispheres [1–6].

Over the past two decades, an increasing number of studies have applied the same tree-ring techniques to the growth increments formed in the hard parts of bivalves and fishes to address analogous issues in the world’s oceans [7–12]. The quality of data is comparable to that of tree-rings in that the same crossdating techniques can be applied to ensure that all increments have been properly identified and assigned the exact calendar year of formation. This ensures the accuracy and precision of the data, and that the final chronologies will capture high-frequency variability, which quickly attenuates with relatively small dating errors [12]. Moreover, the assumption of annual increment formation has been verified in a number of species by sampling stable oxygen isotopes and confirming seasonal cycles [13–17]. Another approach has used the radiocarbon pulse that followed mid-20^th^ century nuclear weapons testing as an independent corroborator of annual growth-increment formation [18,19]. Ultimately, the absolute dating of these growth-increment datasets facilitates their direct inter-comparison to one another as well as comparison with observational environmental records.

Given the extraordinary longevity of some species, notably *Arctica islandica*, high-resolution chronologies can span the past several centuries, and even over the past millennium if dead-collected material is included [20–26]. Growth-increment width can be used as a proxy, though chemical and isotopic compositions can also be sampled to capture a broader array of environmental signals [23]. For *Arctica islandica* in the North Atlantic Ocean, oxygen isotopic composition in the growth increment provides a stronger record of ocean temperature and salinity than width and has been used to reconstruct ocean variability over the last millennium [27]. In addition, carbon isotopic composition has been used to reconstruct the Suess effect of anthropogenic carbon input as well as the carbon reservoir effect [28–30]. Beyond climate reconstructions, growth-increment width is a biological indicator that can correlate with primary production [31] or other indicators of ecosystem function and productivity [32]. As such, sclerochronologies yield baseline information of the climate drivers of biological processes in marine systems, which are often lacking due to the expense of repeated measures in traditional sampling regimes.

The diversity and geography of species used for sclerochronological studies continues to expand, and in the North Atlantic region includes such species as *Glossus humanus* [33], *Mercenaries stimpsoni* [34], *Glycymeris glycymeris* [30,35] *Cyclocardium* spp. and *Serripes* spp [36]. These studies indicate that growth-increment width chronologies respond to different environmental variables among species and sites. Although temperature and availability of food are among the most important [14], the seasonality of this sensitivity can vary [37,38]. This information highlights the potential diversity of climate drivers on biology and also opens the possibility for multi-proxy reconstructions. Ideally, the combination of multiple records would capture a more robust, broader spectrum of signals than could be captured from a single record. As the number of marine chronologies grows, the multi-proxy approaches are increasingly possible [7,39].

**Fig 1:**
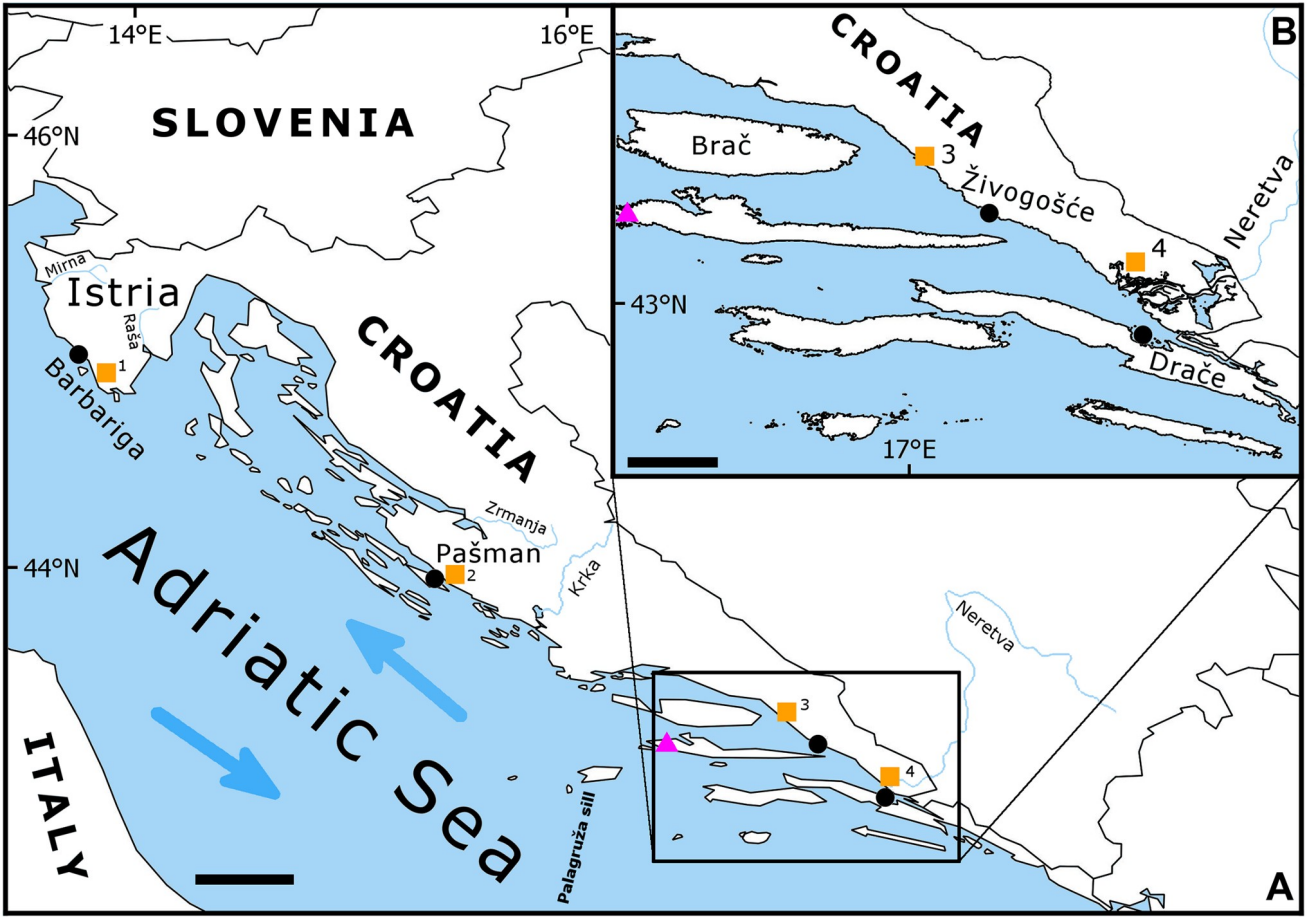
Map of the study area. *Glycymeris pilosa* sampling sites indicated with black points: Istria (Barbariga), Pašman, Živogošće and Drače. Locations of meteorological stations from which air temperature and precipitation data were obtained indicated by orange squares: 1 Pula, 2 Biograd, 3 Makarska and 4 Ploče. Location of station Hvar, from where sea surface temperature data were taken, indicated by pink triangle. Scale bars 50 km (A) and 20 km (B).

To date, the majority of bivalve sclerochronologies have been developed at relatively high latitudes and in major ocean basins. Yet these approaches are possible for semi-enclosed temperate seas, such as the Mediterranean and the Adriatic Seas (Fig 1). Here, chronologies tend to span a few decades due to relatively short longevities [40–42], but are environmentally sensitive and tend to involve some combination of local and remote effects. An example is *Glycymeris pilosa* in the shallow northernmost part of the Adriatic Sea, whose growth has been found to correlate with a decadal (5–10 years) oscillation of water masses [42] referred to as the Adriatic-Ionian Bimodal Oscillating System (BiOS) [43,44]. BiOS is known to be the dominant driver of the thermohaline and biogeochemical oscillations across most of the basin [45–48]. Nearshore and shallow regions, however, might be strongly impacted by coastal processes such as river discharge, especially in the northernmost part of the Adriatic where flows are greatest [49–51]. In the present study, chronologies for a single bivalve species, *G*. *pilosa*, are developed at four locations along the eastern Adriatic. Covariance among chronologies is investigated, as are the relative influences of regional (BiOS) and local (temperature and freshwater input) drivers to better quantify the diversity of climate responses across the region and the potential for multi-proxy reconstructions.

## Materials and methods

### Shell collection and preparation

*Glycymeris pilosa* samples were live collected by SCUBA divers from two locations in the eastern Adriatic: Živogošće (3–5 m depth, 43°10'57.02"N, 17° 9'42.91"E), and Drače (4–5 m depth, 42°56'10.54"N, 17°28'23.71"E) (Fig 1). Permit for the field research was obtained from the Ministry of the Sea, Transportation and Infrastructure of the Republic of Croatia. Field study did not involve endangered or protected species. In order to collect a sufficient number of large-sized shells, sampling was conducted on several occasions. At Živogošće, sampling took place in June 2015, and June, September, and October 2016. At Drače, sampling was conducted in July 2015 and July 2016. After collection, specimens were transported to the laboratory where soft tissues were carefully removed and the shells then washed, air dried, and measured for length and weight. The largest shells (>60 mm) were selected for sclerochronological analysis.

The analysed specimens from Živogošće ranged in shell length from 61.1 to 88.0 mm (74.2±5.3 mm; N = 44), and their weights ranged from 86.8 to 240.0 g (144.4±36.8 g). The analysed shells from Drače ranged in length from 61.0 to 86.2 mm (71.6±5.5 mm; N = 50), and in weight from 86.1 to 310.0 g (148.2±47.4 g). Samples were processed following procedures described in Peharda et al. [41,42]. The hinge area was cut from the shell using a Struers Labotom 3 saw and then embedded in epoxy resin. Each resin block was cut along the axis of maximum shell growth, after which the exposed surface was ground on a series of wettened silicon carbide papers (220–2000 grit) and polished using a soft cloth impregnated with diamond paste (3 μm). Shell sections were etched in 0.1 M HCl for 2–3 min or in 0.3 M HCl for 30 s and acetate peels were prepared. Acetate peel images were photographed using an Axio Lab A1 microscope equipped with a Zeiss AxioCam ERc 5s camera. Multiple photomicrographs of each sample were taken, and then stitched into single panoramic using Image-Pro Premier software (Media Cybernetics, Silver Spring, MD, USA).

### Growth increment measurements and chronology construction

For each site, all samples were visually crossdated by matching synchronous growth patterns induced by environmental variability among shells [52,53]. The procedure began with the increment formed at the known year of capture, working backward in time toward the innermost increment formed during the first year of life. Once visually crossdated, growth-increment widths were measured continuously along the axis of maximum growth using Image-Pro Premier software (Media Cybernetics, Silver Spring MD, USA). Quantitative verification of crossdating was conducted in the program COFECHA [54] in which each measurement time series was fitted with its own 15-year 50% frequency cut-off cubic smoothing spline. Observed values were divided by those predicted to isolate high-frequency patterns of growth variability after which each detrended time series was correlated with the mean of all others. COFECHA was used to calculate series intercorrelation, the mean correlation between each standardized time series and the average of all others, and mean sensitivity [55], an index of high frequency (year-to-year) variability between pairs of successive increments.

The standard chronology was constructed in the software package ARSTAN [56] using the original measurement time series. Each measurement time series was adaptive first power transformed [57], and then detrended by calculating the residuals (differences) between each set of power-transformed measurements its best-fit negative exponential or regression function.

Chronologies are generally truncated where Subsample Signal Strength (SSS), a measure of common signal shared among samples, consistently falls below a threshold of 0.85. As sample size diminishes back through time, so does the strength of this common signal. An SSS > 0.85 ensures that at least 85% of this common signal is represented in the chronology and that the sample depth is therefore adequate. Although arbitrary and lacking statistical significance, and SSS threshold of 0.85 is considered adequate for establishing climate reconstructions or climate-growth relationships [58]. The SSS statistic, however, is calculated in a 50-year running window, which is shortened here to a 30-year running window to accommodate the brevity of the measurement time series. Given the temporal smoothing induced by this running window, an exact date at which SSS thresholds are crossed cannot be determined. Thus, the chronologies are truncated at a sample depth of ten, which is a conservative estimate at which SSS values are generally robust across all chronologies. The low-frequency component of the chronology was highlighted by fitting a 20-year 50% frequency cut-off cubic smoothing spline to each chronology.

### Chronology comparison and environmental analysis

*G*. *pilosa* chronologies from Živogošće and Drače were compared to previously published *G*. *pilosa* chronologies from the northern and eastern Adriatic Sea: Istria (10–11 m, 44°59′7.47″N, 13°44′19.22″E) [42] and Pašman Channel (2–3 m depth, 43°56'52.68"N, 15°23'15.03"E) [41].

The chronologies were related to monthly-averaged environmental indices using Spearman correlation, the significance of which was assessed by a bootstrapped confidence interval using the R package TreeClim [59]. Air temperature and precipitation data were obtained from the Croatian Meteorological and Hydrological Service at the closest meteorological stations (Pula, Biograd, Makarska, and Ploče for Istria, Pašman, Živogošće and Drače chronologies, respectively). For two southern chronologies, sea surface temperature data were taken from station Hvar, which is 60 km west of the Živogošće sampling location. Regimes of the Adriatic-Ionian Bimodal Oscillating System (BiOS) have been quantified by Absolute Dynamic Topography ADT variable, which is defined as the difference between satellite-derived absolute dynamic topography at the perimeter of the northern Ionian gyre (39.2–39.8°N, 18.8–19.2°E) and its centre (38.0–38.6°N, 18.4–18.8°E). These data have been available since 1993 from Copernicus—Marine Environment Monitoring Service (http://marine.copernicus.eu/). Positive ADT indicates cyclonic BiOS regime and circulation in the northern Ionian gyre, advecting saline and ultraoligothophic waters to the Adriatic from the Eastern Mediterranean while negative ADT indicates anticyclonic BiOS regime and circulation in the northern Ionian gyre, advecting less saline and nutrient-richer waters from the Western Mediterranean [43,60]. Monthly climate data were used given that conditions during a certain season may influence the entire year of growth, often by influencing growing-season length. These correlations were assessed over all months of the current year as well as those in the prior year to quantify any lagged effects.

Monthly mean flow rates of the river Neretva were compared to the Živogošće and Drače chronologies using Spearman correlations. Average monthly river flow data (m^3^/s) at the Žitomislići (Bosnia and Herzegovina) station were available from January 1980 to December 1990, and from January 2004 to December 2013. No substantial freshwater discharges are present close to the northern sampling sites at Istria and Pašman [61].

## Results

### Živogošće and Drače chronologies

Growth increments were clearly visible in acetate peels of 20 shells from the Živogošće sampling site, and 29 shells from the Drače sampling site. Estimated ages of individuals from Živogošće ranged from 33 to 61 years (46.5±8.5 years), while that from Drače ranged from 31 to 97 years (52.7±12.2 years). A total of 7 shells from Živogošće had estimated age of over 50 years, while at Drače 16 shells had estimated age of over 50 years.

**Fig 2:**
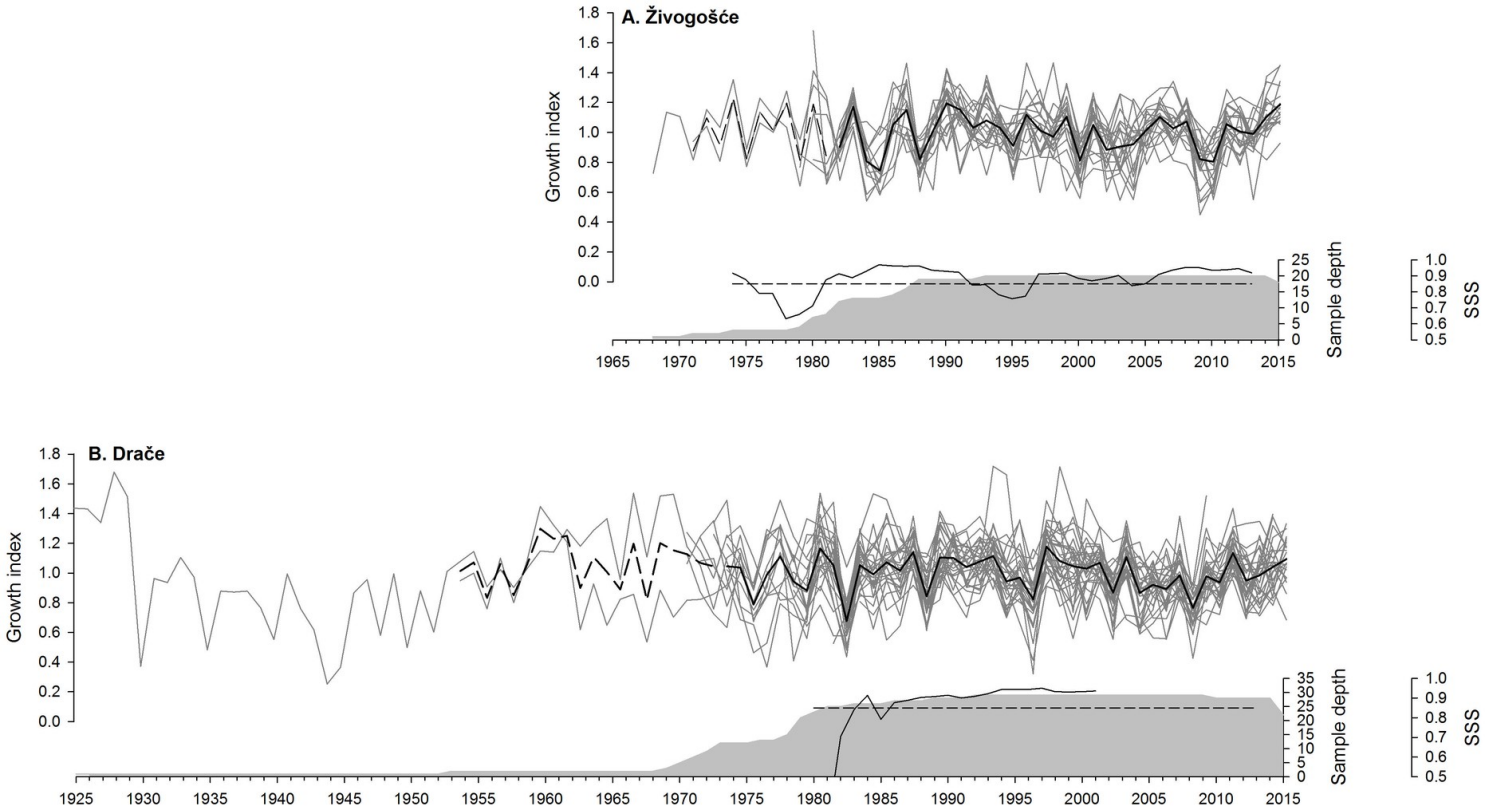
*Glycymeris pilosa* chronology. **A**. Individual detrended growth time series of shells from Živogošće (1968–2015) and their average (1982 statistically robust chronology in black, prior to 1982 in dashed line), **B**. Individual detrended growth time series of shells from Drače (1924–2015) and their average (1973 statistically robust chronology in black, prior to 1973 in dashed line). Sample depth (denoting number of samples, grey shading area), 0.85 Subsample Signal Strength (SSS) limit (straight dashed line) and calculated SSS (black line) presented for each sampling site below corresponding chronology data.

The mean correlation between each detrended time series and the average of others (series intercorrelation) for shells from Živogošće was 0.563, while average mean sensitivity was 0.195. For shells from Drače, the series intercorrelation was 0.582 and mean sensitivity was 0.205. The oldest measured growth increment from Živogošće dates back to year 1968, while at Drače dates back to the year 1924, resulting in 91 years of data through 2015 (Fig 2A).

Synchronous growth patterns were observed within each location as evidenced by visual crossdating. At Živogošće, narrow growth increments occurred in 2010, 2009, 2000, 1988, and 1985, while wider growth increments occurred in 2015, 2006, and 1990 (Fig 2B). At Drače, conspicuously narrow growth increments corresponded to years 2008, 1996, 1988, and 1982, while wide ones corresponded to 2011, 2003, and 1997. According to the subsample signal strength and sample depth, the Živogošće chronology is robust from 1982, spanning a total of 33 years, while the Drače chronology is robust from 1973, spanning a total of 42 years.

### Chronology network

**Fig 3:**
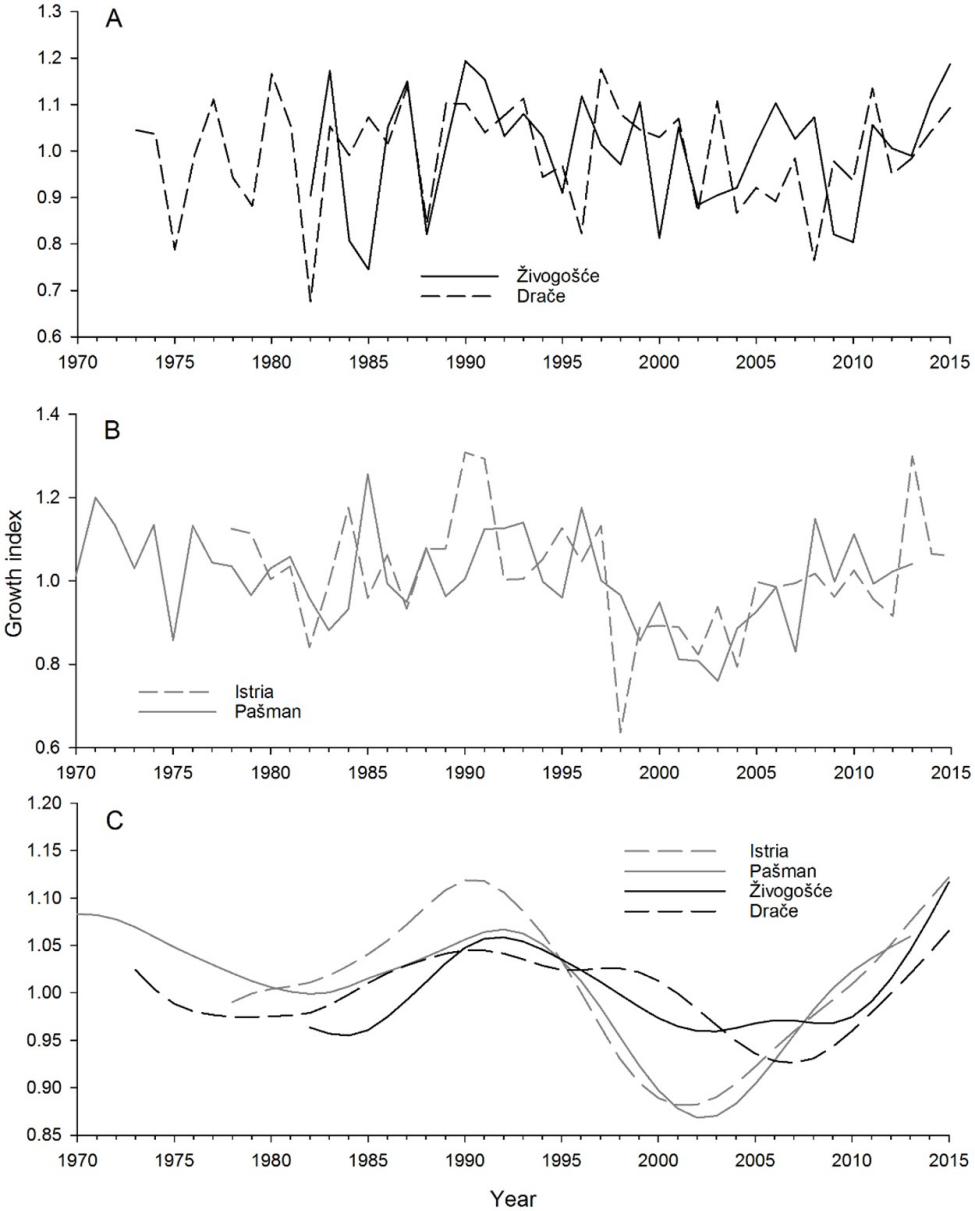
Comparison of *Glycymeris pilosa* shell growth chronologies. **A**. Živogošće and Drače (this study), **B**. Istria (from [15]) and Pašman (from [41]), **C**. The low-frequency component of all four chronologies as modelled by a 20-year 50% frequency cutoff cubic smoothing spline fit to each chronology.

**Fig 4:**
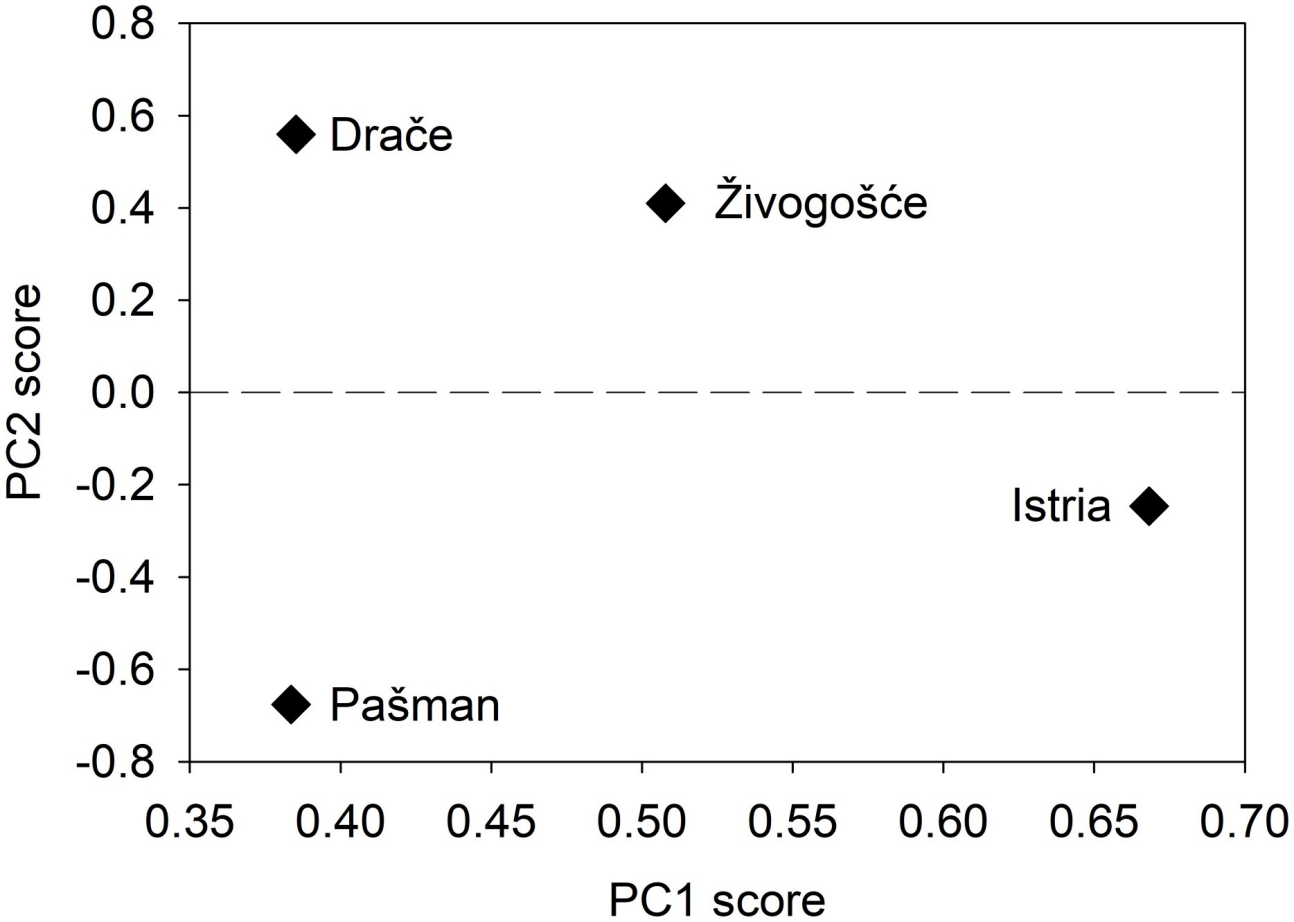
Scores for axes 1 and 2 in a principal components analysis of the four *Glycymeris pilosa* chronologies: Istria, Pašman, Živogošće and Drače.

Chronologies obtained for *Glycymeris pilosa* shells from Živogošće and Drače were weakly and positively correlated (r = 0.304, p = 0.080, N = 34) (Fig 3A). Narrow growth increments were observed in years 1988 and 2002 at both locations, while wide growth increments were noted in 1987, 2011 and 2015. Živogošće and Drače chronologies were also not significantly related to two previously published *G*. *pilosa* chronologies from two northern sites in the Adriatic Sea—Pašman [41] and a shallow embayment of the northern Adriatic Sea (Istria) [42]. For Pašman, Spearman correlation coefficients with Živogošće and Drače chronologies were 0.084 (p = 0.647; N = 32) and 0.042 (p = 0.796; N = 41), respectively. For Istria, these values were 0.215 (p = 0.222, N = 34) and 0.032 (p = 0.848, N = 38), respectively. The two northern chronologies had moderately strong correlations with each other (r = 0.465, p = 0.004, N = 36; Fig 3A), and this correlation appeared to be mostly driven by a low-frequency signal (Fig 3C). Overall, there is no clear grouping of these four chronologies in a principal components analysis that spans their common interval (Fig 4). Principal component (PC) 1 explained 36% of the variability in the dataset while PC2 explained an additional 31%. Only these two PCs were retained given that there each had an eigenvalue greater than one. Ordering along the second principal component loosely followed latitude, though Istria and Pašman are inverted along this gradient.

### Environmental associations

**Fig 5:**
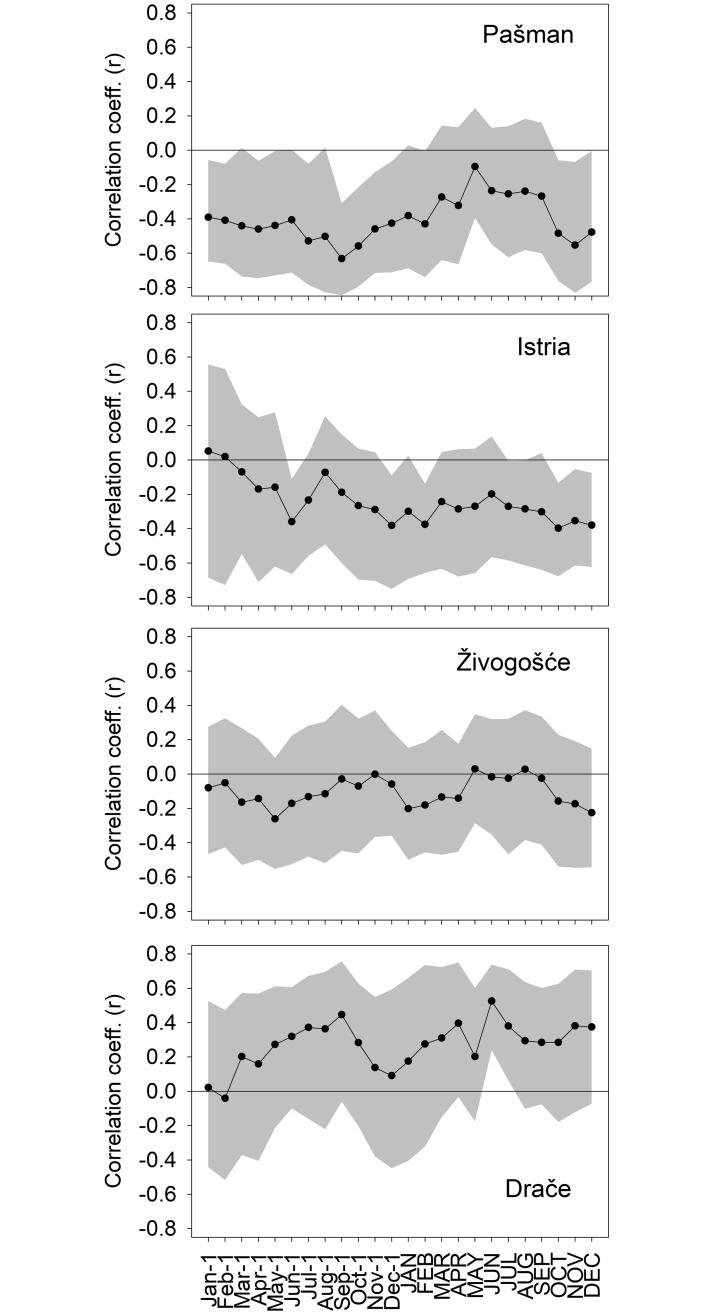
Correlations between each of the four chronologies and monthly-averaged ADT over the current and prior year. Grey shading is 95% confidence intervals from a bootstrapped confidence test. Months are significant (p<0.05) if the confidence interval (grey shading) does not cross zero.

Neither the Živogošće nor the Drače chronologies significantly related to records of air temperatures, sea water temperature or precipitation. The Pašman and Istria chronologies both correlated strongly to sea water temperature data as has been previously published for these northern two chronologies [41,42]. The correlations between chronology and ADT varied along a latitudinal gradient from negative relationships in the north to positive relationships in the south and neutral, non-significant relationships in the mid-range of the sampling region (Fig 5). The seasonality of ADT-growth relationships also contrasted between the northern and southern sites. To the north, relationships were significant through much of the prior year as well as the cool season of the current year. By contrast at the southernmost site of Drače, significant relationships occurred in a narrow window of the current warm season (Fig 5).

**Fig 6:**
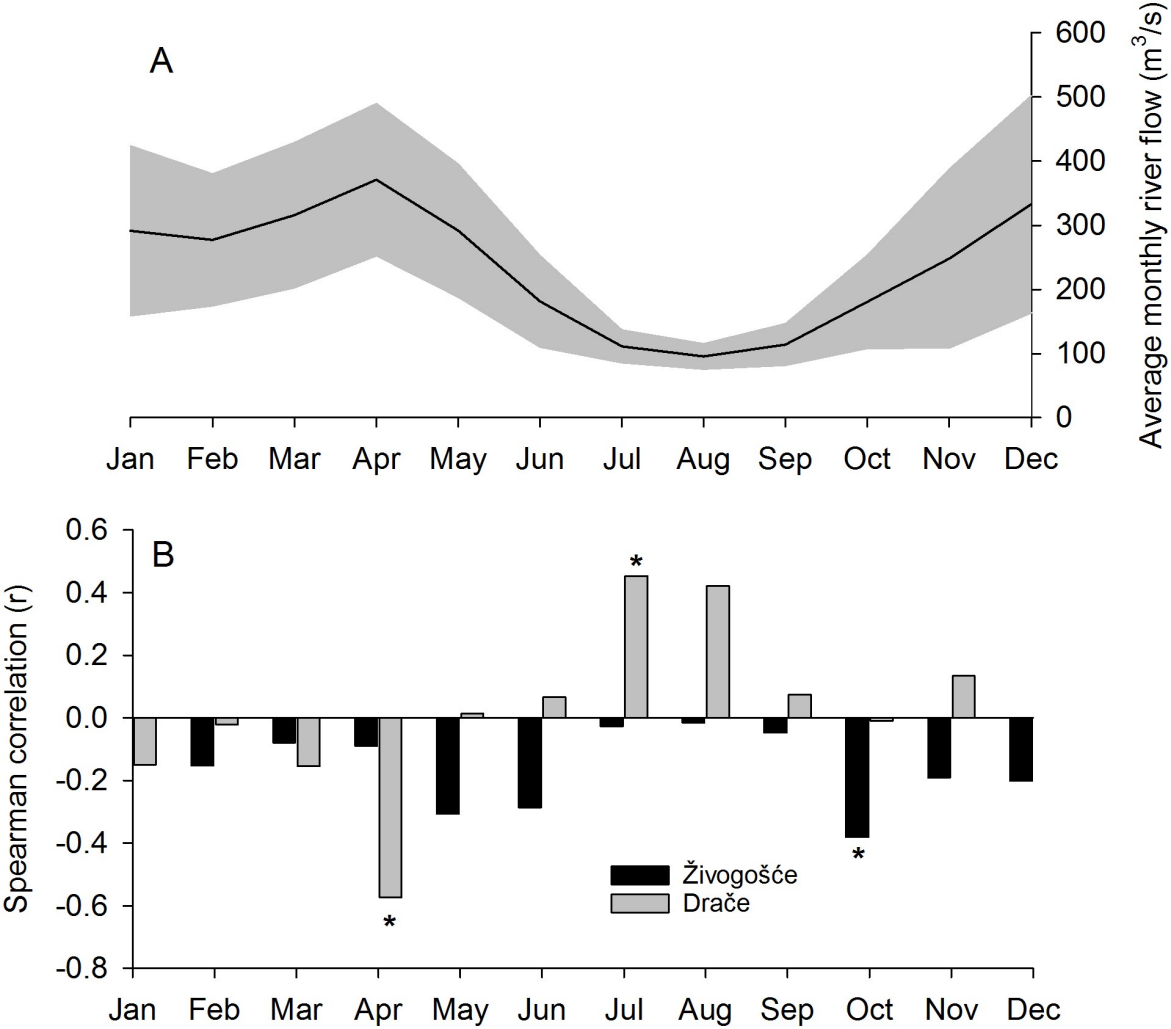
**A**. Average monthly river flow rate (in m^3^/s) of river Neretva at station Žitomislići (Bosnia and Herzegovina), black line indicates average values for 1980–1990 and 2004–2013 period, shaded areas represent ± one standard deviation. **B**. Spearman correlation values between shell chronologies for Živogošće and Drače and average monthly flow rates for Neretva. Significant values (p<0.05) are indicated with asterix.

A significant negative correlation was noted between the *G*. *pilosa* chronology for Drače and average monthly river flow of Neretva in April (r = -0.573, p<0.05; Fig 6). April is the month with the highest average monthly discharge values, reaching 371 m^3^/s. A positive correlation between Drače chronology and Neretva flow was also found for month of July (r = 0.453, p < 0.05), which with August and September have the lowest average river flow values (111, 96 and 114 m^3^/s, respectively). The Živogošće chronology was significantly and negatively correlated with Neretva flow in the month of October (r = -0.381, p<0.05).

## Discussion and conclusion

### Živogošće and Drače chronologies

The two chronologies developed here further corroborate that *Glycymeris pilosa* growth is synchronous within populations [41,42], highlighting the potential for networks of crossdated, annually-resolved chronologies for the Adriatic Sea. The four chronologies developed to date span a latitudinal gradient of approximately 375 km. The Živogošće chronology extended for more than three decades, from the early 1980s to the mid 2010s, and is a decade shorter than the Drače (this study), Pašman Channel [41] and Istria chronologies [42]. The relatively short timespan at Živogošće can be attributed to a smaller sample size and the longevity of analysed shells, which largely did not pre-date the 1980s. Although the maximum longevity of *G*. *pilosa* was 97 years, the overall scarcity of such old individuals suggests that the potential of constructing robust *G*. *pilosa* chronologies spanning more than five decades is a challenge.

### Bivalve chronology networks

Networks of marine shell-based chronologies have been developed for several species including *Panopea generosa* from the northeastern Pacific [7], *Arctica islandica* from the North Sea [62], and *Glycymeris glycymeris* from the Irish Sea [63]. In some cases, such as *Panopea generosa* and *Glycymeris glycymeris*, climate-growth relationships are consistent across sites. Growth rates for both of these species have been found to respond to water temperature. However, this does not necessarily translate to coherence in growth across the chronology networks due to the heterogeneity of local climate regimes. This is particularly true for coastal regions where local climate can vary across relatively short spatial scales. In our study, the four *G*. *pilosa* populations from the Adriatic Sea did not correlate, reflecting potentially heterogenous climate regimes along the coast or different limiting environmental factors along this longitudinal gradient of the Adriatic Sea. The results of this study suggest it may be a combination of climate heterogeneity and contrasting climate drivers.

### Drivers of interannual variability in shell growth

The latitudinal gradient of the four chronologies captures a range of potential environmental drivers important to shell growth. Drače and Živogošće are located in the coastal eastern middle Adriatic where the Neretva River discharge decreases the salinity and delivers a substantial amount of nutrients to coastal waters [64,65]. This region may also be protected from broader-scale patterns of oceanic circulation. The Eastern Adriatic Current (EAC), which brings water masses from the Eastern Mediterranean and the Ionian Sea to the northern Adriatic [51,66], is forced to cross the Palagruža Sill far from the eastern coastal region. The crossing is determined by bathymetry constrictions, i.e. by middle Adriatic islands reducing the transport between coastal middle Adriatic and open sea [67]. Thus, river discharge is likely to influence biology in the coastal eastern middle Adriatic as reflected in the climate-growth relationships of Drače and Živogošće.

In contrast, the two northern sites, Istria and Pašman, are exposed to EAC-driven inflow of saline waters from the southeast and experience relatively low local freshwater inputs, while large northern Adriatic rivers flow along the western Adriatic shoreline [51]. Salinity at these sites is therefore relatively high [68]. Even though Pašman site is located inside the channels, there are no major river influences on the thermohaline and biogeochemical properties [62]. Thermohaline and biogeochemical variability of the southern and middle Adriatic is well connected with the BiOS (e.g. [45,60,69]) as is the shallow northern Adriatic [42,48]. It might be reasonably assumed that the BiOS is the major driver of interannual variability in shell growth along the majority of the eastern Adriatic coastline, aside from where freshwater inputs over-ride these signals as may be the case along the coastal middle Adriatic as represented by Živogošće and Drače.

Seawater temperature controls the seasonal growth of *G*. *pilosa* in the Adriatic Sea, which slows and/or ceases during colder part of the year [15] whilst growing vigorously during summer. However, relationships between temperature and interannual growth are not particularly strong for these Adriatic chronologies, especially those in the southern part of the study region. The strongest correlate overall is with ADT variable representing BiOS (Fig 5), particularly at the northern sites, which underscores the importance of this broad-scale, low-frequency oceanographic process to the biology of the system. It might appear that an increased inflow of waters poor or richer in nutrients from the Eastern or Western Mediterranean during the respective BiOS regime (cyclonic vs. anticyclonic) and the consequent change in nutrient availability and primary production rate in the whole Adriatic is also affecting coastal areas less exposed to local nutrient load by freshwater discharges, here Istria and Pašman sampling sites. Furthermore, the temperature of waters coming from the Eastern Mediterranean are slightly higher than of the Western Mediterranean [46], thus changing the accumulated heat in the whole Adriatic, including coastal sites not strongly affected by local processes. Yet, local sea temperature in the surface layer is strongly under the effect of direct heating from the atmosphere, so quantification of effects to the growth of these two processes—the direct heating and advection of heat by BiOS—remains unknown and needs further investigation.

For the northern two chronologies, temperature may at least partially be involved. However, it is likely that unmeasured variables such as nutrients and food supply are also likely important [70–72]. Moreover, the environmental variables that influence growth may vary over fine spatial scales. This is especially true for coastal environments in semi enclosed seas, such as the Adriatic, where local processes, including precipitation, river and terrestrial runoff, may be highly localized [73]. Thus, the fact that shells crossdate within sites indicates that growth is responding to some shared environmental signal(s), but the relatively weak correlations between chronology and climate suggests that observational records for these drivers are lacking. On a broader scale, adding shell chronologies from the western coastline would better demonstrate the importance of BiOS to the broader Adriatic. In contrast to the eastern Adriatic shoreline, the western Adriatic shoreline is strongly influenced by the Western Adriatic Current (WAC) [51], which carries freshwater inputs and associated nutrients from the northern Adriatic and thereby enhances primary production [74]. With this circulation pattern, the hypothesis is that BiOS may be well correlated to shell growth on the western coastlines.

Although longevity of *Glycymeris pilosa* can approach a century, such long-lived individuals appear to be uncommon. Though there may ultimately prove to be exceptions, chronologies do not substantially pre-date instrumental records or enable multi-centennial paleoclimate reconstruction. However, from an ecological perspective, better understanding heterogeneity in climate and biological response may provide insight into the resilience of the Adriatic coastal ecosystems. A greater diversity or “portfolio” of climate responses may increase ecosystem resilience. Should an extreme environmental event occur, not all components or locations of the ecosystem may respond identically such that at least some individuals are likely to be less negatively impacted to “rescue” those in subpopulations that are. Such diversity in climate response may also complicate attempts to disentangle climate from human impacts. A higher density of sites may provide a stronger baseline for these environmental impacts. At the same time, these shells provide key information on environmental drivers of biological processes, with for example the relative importance of BiOS vs. fine-scale, local variability. Thus, *G*. *pilosa* provides a uniquely long window into biological processes and is broadly distributed throughout the region. Finally, Also, shell-derived chronologies capture variability in coastal zones where variability in environmental drivers is often quite high. Therefore, a broader network of multidecadal chronologies, which are periodically updated, could serve a wide range of roles in quantifying the ecology and environmental drivers of a semi-enclosed sea, here the Adriatic Sea.

## Supporting information

S1 File (XLSX)
